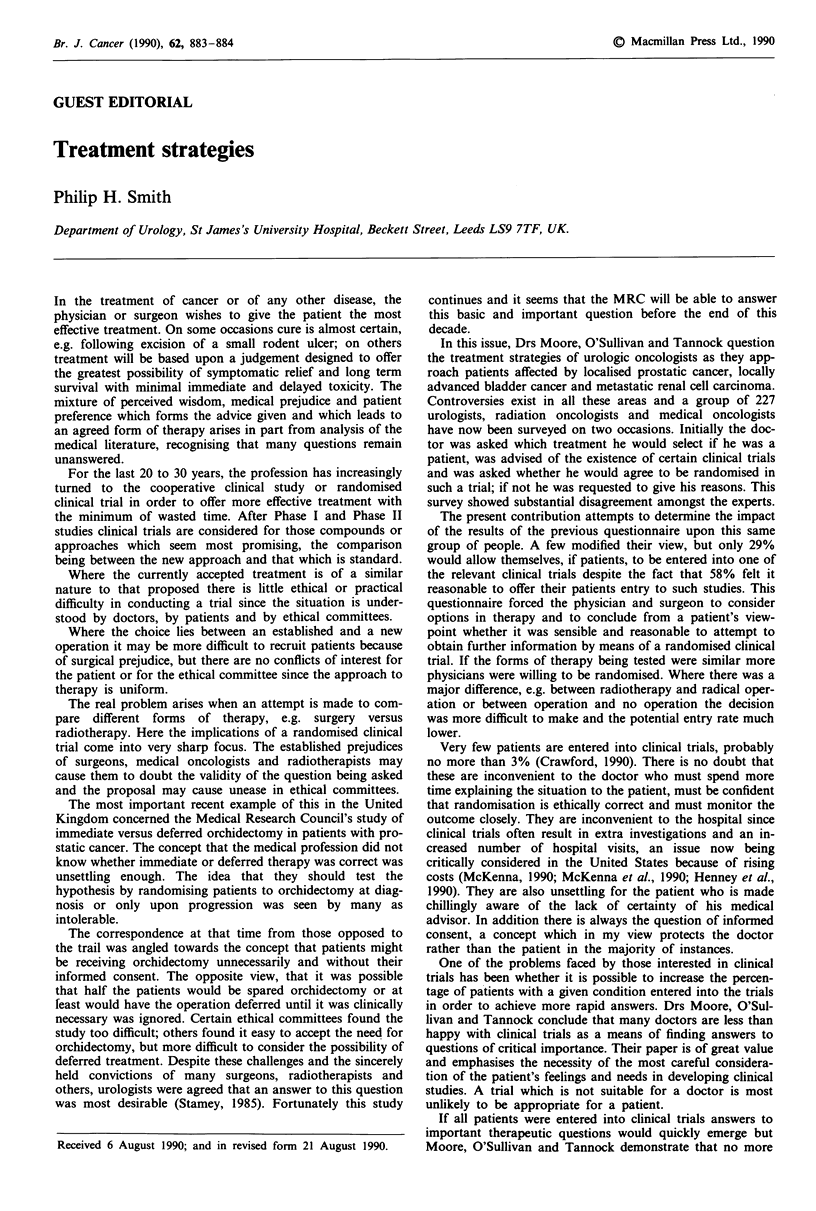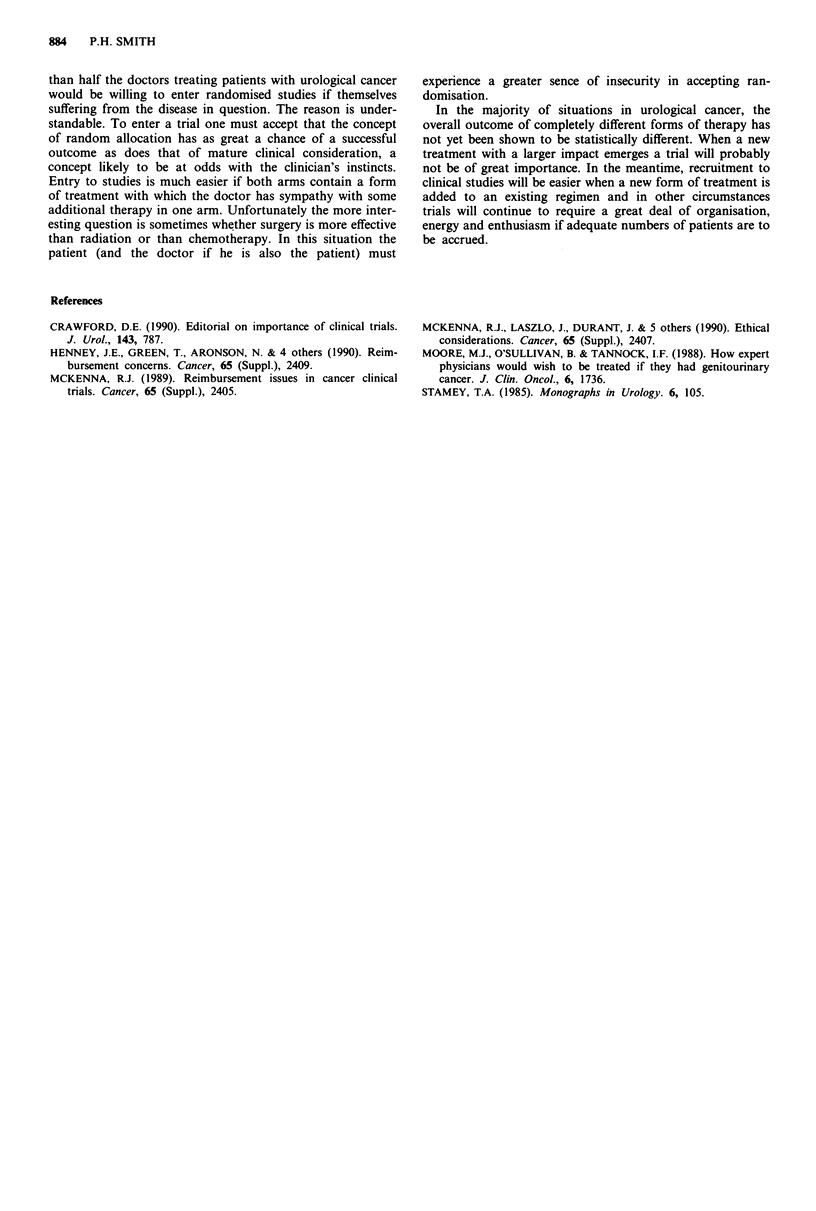# Treatment strategies.

**DOI:** 10.1038/bjc.1990.402

**Published:** 1990-12

**Authors:** P. H. Smith


Br. J. Cancer (1990), 62, 883-884 © Macmillan Press Ltd., 1990

GUEST EDITORIAL

### Treatment strategies

Philip H. Smith

*Department of Urology, St James's University Hospital, Beckett Street, Leeds LS9 7TF, UK.*

In the treatment of cancer or of any other disease, the physician or surgeon wishes to give the patient the most effective treatment. On some occasions cure is almost certain, e.g. following excision of a small rodent ulcer; on others treatment will be based upon a judgement designed to offer the greatest possibility of symptomatic relief and long term survival with minimal immediate and delayed toxicity. The mixture of perceived wisdom, medical prejudice and patient preference which forms the advice given and which leads to an agreed form of therapy arises in part from analysis of the medical literature, recognising that many questions remain unanswered.

For the last 20 to 30 years, the profession has increasingly turned to the cooperative clinical study or randomised clinical trial in order to offer more effective treatment with the minimum of wasted time. After Phase I and Phase II studies clinical trials are considered for those compounds or approaches which seem most promising, the comparison being between the new approach and that which is standard.

Where the currently accepted treatment is of a similar nature to that proposed there is little ethical or practical difficulty in conducting a trial since the situation is understood by doctors, by patients and by ethical committees.

Where the choice lies between an established and a new operation it may be more difficult to recruit patients because of surgical prejudice, but there are no conflicts of interest for the patient or for the ethical committee since the approach to therapy is uniform.

The real problem arises when an attempt is made to compare different forms of therapy, e.g. surgery versus radiotherapy. Here the implications of a randomised clinical trial come into very sharp focus. The established prejudices of surgeons, medical oncologists and radiotherapists may cause them to doubt the validity of the question being asked and the proposal may cause unease in ethical committees.

The most important recent example of this in the United Kingdom concerned the Medical Research Council's study of immediate versus deferred orchidectomy in patients with prostatic cancer. The concept that the medical profession did not know whether immediate or deferred therapy was correct was unsettling enough. The idea that they should test the hypothesis by randomising patients to orchidectomy at diagnosis or only upon progression was seen by many as intolerable.

The correspondence at that time from those opposed to the trail was angled towards the concept that patients might be receiving orchidectomy unnecessarily and without their informed consent. The opposite view, that it was possible that half the patients would be spared orchidectomy or at least would have the operation deferred until it was clinically necessary was ignored. Certain ethical committees found the study too difficult; others found it easy to accept the need for orchidectomy, but more difficult to consider the possibility of deferred treatment. Despite these challenges and the sincerely held convictions of many surgeons, radiotherapists and others, urologists were agreed that an answer to this question was most desirable (Stamey, 1985). Fortunately this study continues and it seems that the MRC will be able to answer this basic and important question before the end of this decade.

In this issue, Drs Moore, O'Sullivan and Tannock question the treatment strategies of urologic oncologists as they approach patients affected by localised prostatic cancer, locally advanced bladder cancer and metastatic renal cell carcinoma. Controversies exist in all these areas and a group of 227 urologists, radiation oncologists and medical oncologists have now been surveyed on two occasions. Initially the doctor was asked which treatment he would select if he was a patient, was advised of the existence of certain clinical trials and was asked whether he would agree to be randomised in such a trial; if not he was requested to give his reasons. This survey showed substantial disagreement amongst the experts.

The present contribution attempts to determine the impact of the results of the previous questionnaire upon this same group of people. A few modified their view, but only 29% would allow themselves, if patients, to be entered into one of the relevant clinical trials despite the fact that 58% felt it reasonable to offer their patients entry to such studies. This questionnaire forced the physician and surgeon to consider options in therapy and to conclude from a patient's viewpoint whether it was sensible and reasonable to attempt to obtain further information by means of a randomised clinical trial. If the forms of therapy being tested were similar more physicians were willing to be randomised. Where there was a major difference, e.g. between radiotherapy and radical operation or between operation and no operation the decision was more difficult to make and the potential entry rate much lower.

Very few patients are entered into clinical trials, probably no more than 3% (Crawford, 1990). There is no doubt that these are inconvenient to the doctor who must spend more time explaining the situation to the patient, must be confident that randomisation is ethically correct and must monitor the outcome closely. They are inconvenient to the hospital since clinical trials often result in extra investigations and an increased number of hospital visits, an issue now being critically considered in the United States because of rising costs (McKenna, 1990; McKenna *et al.*, 1990; Henney *et al.*, 1990). They are also unsettling for the patient who is made chillingly aware of the lack of certainty of his medical advisor. In addition there is always the question of informed consent, a concept which in my view protects the doctor rather than the patient in the majority of instances.

One of the problems faced by those interested in clinical trials has been whether it is possible to increase the percentage of patients with a given condition entered into the trials in order to achieve more rapid answers. Drs Moore, O'Sullivan and Tannock conclude that many doctors are less than happy with clinical trials as a means of finding answers to questions of critical importance. Their paper is of great value and emphasises the necessity of the most careful consideration of the patient's feelings and needs in developing clinical studies. A trial which is not suitable for a doctor is most unlikely to be appropriate for a patient.

If all patients were entered into clinical trials answers to important therapeutic questions would quickly emerge but Moore, O'Sullivan and Tannock demonstrate that no more

Received 6 August 1990; and in revised form 21 August 1990.

than half the doctors treating patients with urological cancer would be willing to enter randomised studies if themselves suffering from the disease in question. The reason is understandable. To enter a trial one must accept that the concept of random allocation has as great a chance of a successful outcome as does that of mature clinical consideration, a concept likely to be at odds with the clinician's instincts. Entry to studies is much easier if both arms contain a form of treatment with which the doctor has sympathy with some additional therapy in one arm. Unfortunately the more interesting question is sometimes whether surgery is more effective than radiation or than chemotherapy. In this situation the patient (and the doctor if he is also the patient) must experience a greater sence of insecurity in accepting randomisation.

In the majority of situations in urological cancer, the overall outcome of completely different forms of therapy has not yet been shown to be statistically different. When a new treatment with a larger impact emerges a trial will probably not be of great importance. In the meantime, recruitment to clinical studies will be easier when a new form of treatment is added to an existing regimen and in other circumstances trials will continue to require a great deal of organisation, energy and enthusiasm if adequate numbers of patients are to be accrued.

**References**

CRAWFORD, D.E. (1990). Editorial on importance of clinical trials. *J. Urol.*, **143**, 787.

HENNEY, J.E., GREEN, T., ARONSON, N. & 4 others (1990). Reimbursement concerns. *Cancer*, **65** (Suppl.), 2409.

MCKENNA, R.J. (1989). Reimbursement issues in cancer clinical trials. *Cancer*, **65** (Suppl.), 2405.

MCKENNA, R.J., LASZLO, J., DURANT, J. & 5 others (1990). Ethical considerations. *Cancer*, **65** (Suppl.), 2407.

MOORE, M.J., O'SULLIVAN, B. & TANNOCK, I.F. (1988). How expert physicians would wish to be treated if they had genitourinary cancer. *J. Clin. Oncol.*, **6**, 1736.

STAMEY, T.A. (1985). *Monographs in Urology*. **6**, 105.